# BRCA1 polymorphism in breast cancer patients from Argentina

**DOI:** 10.3892/ol.2014.2772

**Published:** 2014-12-05

**Authors:** OMAR JAURE, ELIANA N. ALONSO, DIEGO AGUILERA BRAICO, ALVARO NIETO, MANUELA OROZCO, CECILIA MORELLI, ALEJANDRO M. FERRO, ELENA BARUTTA, ESTEBAN VINCENT, DOMINGO MARTÍNEZ, IGNACIO MARTÍNEZ, MARIA INES MAEGLI, ALEJANDRO FRIZZA, RUBEN KOWALYZYN, MARISA SALVADORI, PAUL GINESTET, MARIA L. GONZALEZ DONNA, GABRIELA A. BALOGH

**Affiliations:** 1 Biotechnology Laboratory, Center of Research and Technology, CERZOS-CONICET, Bahía Blanca, Buenos Aires 8000, Argentina; 2 South Regional Italian Hospital, Bahía Blanca, Buenos Aires 8000, Argentina; 3 Female Medical Institute MEDIFEM, Dr Leonidas Lucero’s Hospital, Bahía Blanca, Buenos Aires 8000, Argentina; 4 Lavalle Institute of Diagnostics, Dr Leonidas Lucero’s Hospital, Bahía Blanca, Buenos Aires 8000, Argentina; 5 Gynecology Services, Dr Leonidas Lucero’s Hospital, Bahía Blanca, Buenos Aires 8000, Argentina; 6 Viedma Clinic, Viedma, Rio Negro 8500, Argentina; 7 Lucio Molas’s Hospita, Santa Rosa, La Pampa 6300, Argentina; 8 Pigue Municipal’s Hospital, Pigue, Buenos Aires 8170, Argentina

**Keywords:** BRCA1, breast cancer, polymorphisms

## Abstract

Breast cancer is the most common type of cancer in females in Argentina, with an incidence rate similar to that in the USA. However, the contribution of the BRCA1 or BRCA2 mutation in breast cancer incidence has not yet been investigated in Argentina. In order to evaluate which BRCA1 polymorphisms or mutations characterize female breast cancer in Argentina, the current study enrolled 206 females with breast cancer from several hospitals from the southeast of Argentina. A buccal smear sample was obtained in duplicate from each patient and the DNA samples were processed for polymorphism analysis using the single-strand conformational polymorphism technique. The polymorphisms in BRCA1 were investigated using a combination of 15 primers to analyze exons 2, 3, 5, 20 and 11 (including the 11.1 to 11.12 regions). The BRCA1 mutations were confirmed by direct sequencing. Samples were successfully examined from 154 females and, among these, 16 mutations were identified in the BRCA1 gene representing 13.9% of the samples analyzed. One patient was identified with a polymorphism in exon 2 (0.86%), four in exon 20 (3.48%), four in exon 11.3 (3.48%), one in exon 11.7 (0.86%), two in exon 11.8 (1.74%), one in exon 11.10 (0.86%) and one in exon 11.11 (0.86%). The most prevalent alteration in BRCA1 was located in exon 11 (11 out of 16 patients; 68.75%). The objective of our next study is to evaluate the prevalence of mutations in the BRCA2 gene and analyze the BRCA1 gene in the healthy relatives of BRCA1 mutation carriers.

## Introduction

Breast cancer is the most prevalent and leading cause of mortality among females worldwide. Through epidemiological studies, it has been found that family history is one of the major risk factors that increases susceptibility to breast cancer. Approximately 5–10% of breast cancers have a hereditary component (1), and inherited mutations in high penetrance genes, such as BRCA1 and BRCA2 (2), closely correlate with an increased risk of females of different ethnic and age groups developing breast and/or ovarian cancer (1). In addition, such mutations in the germline increase the susceptibility to develop cancer of the colon, prostate, pancreas and melanoma (2). It is evident that these mutations are involved in a small fraction of all cancers detected and the presence of sequence alterations in the BRCA genes has significant clinical relevance.

Mutations in the BRCA1 and BRCA2 genes are transmitted from one generation to the next by an autosomal dominant (3). The germline mutations are usually point mutations (4) and are commonly frameshift small insertions or deletions, non-sense mutations, or mutations affecting the splicing sites (2). The majority of these mutations identified in familial breast cancer result in the partial or complete deletion of exons or intronic sequence inserts, which may ultimately yield non-functional, truncated BRCA1 and BRCA2 proteins (2). Approximately 50% of these mutations are located within exon 11 of BRCA1 and BRCA2 (5). Females who carry mutations in the BRCA1 gene have an 80% chance of developing breast cancer during their lifetime, and a 65% chance of developing a second breast cancer prior to the age of 70 years. Similarly, females with BRCA2 mutations have an 85% chance of developing breast cancer (6).

BRCA1 and BRCA2 are structurally similar and of similar size (100 and 70 Kb, respectively) (4). The two genes are considered to be tumor suppressor genes. BRCA1 is located on chromosome 17 at the q21 position and consists of 22 exons (60% of the gene corresponding to exon 11) that encode a protein of 1,863 amino acids (7,8). Due to its large size, exon 11 is the main target for mutation detection. BRCA2 is located on chromosome 13 at the 12q position and consists of 27 exons that encode a protein of 3,418 amino acids (9,10).

Functionally, BRCA1 and BRCA2 are involved in a multitude of cellular processes, such as transcriptional regulation in response to DNA damage, maintenance of chromosomal stability and the regulation of genes involved in the cell cycle and apoptosis (11).

In total, >600 mutations have been described in BRCA1, while 450 mutations have been described in BRCA2 (6). Approximately 50% of the unique variants detected in BRCA1 and BRCA2, regardless of the polymorphisms, are variants with unknown pathogenic potential and are thus termed unclassified variants (2). It is also possible to identify variants of unknown significance. These are variations in the gene sequence which have not been identified to affect the function of the protein. It is possible that polymorphisms of the two genes may result in loss of protein function and thereby increase the risk of cancer; however, the polymorphisms may also exist without risk (12).

The aim of this study was to differentiate between harmless deleterious mutations and polymorphisms in the BRCA1 gene of 154 females with breast cancer in the city of Bahía Blanca in Argentina, as well as cities in close proximity to Bahía Blanca. To the best of our knowledge, this is the first study in Argentina to detect variations in the BRCA1 gene.

## Materials and methods

### Study approval

The study was approved by the research ethics committees of the South Regional Italian Hospital (Bahía Blanca, Argentina), FEMALE Medical Institute MEDIFEM (Bahía Blanca, Argentina), Lavalle Institute of Diagnostics (Bahía Blanca, Argentina), Dr Leonidas Lucero’s Hospital (Bahía Blanca, Argentina), Viedma Clinic (Viedma, Argentina), Lucio Molas’s Hospital (Santa Rosa, Argentina) and Pigue Municipal’s Hospital (Pigue, Argentina), and written informed consent was provided by all patients prior to voluntarily participating in this study.

### Sampling

For this study, 154 female patients (age range, 38–67 years), with breast cancer at any stage of disease, with or without familial breast cancer history, and with or without cancer treatment, but had undergone breast surgery were included. From each patient, buccal mucosa cells were obtained from the patients via a cheek swab performed by the doctors responsible for the study in their respective hospitals. Samples were collected in duplicate for analysis. To avoid inadequate samples, the patients were required to fast for 6–8 h, have good oral hygiene and to rinse the mouth several times with water prior to sampling. In addition, the administration of drugs prior to sampling was prohibited, to prevent interference with the subsequent analysis. The doctor who performed the sampling used two swabs simultaneously to achieve a duplicate sample. The sterile swabs (Cole-Parmer, Vernon Hills, IL, USA) were removed from their packaging and rubbed firmly and several times on the bilateral buccal mucosa of the patient. The swabs were then air-dried for 15 min, and sent to the laboratory (Breast Cancer Research Laboratory, CERZOS-CONICET, Bahía Blanca, Argentina)within 24 h. The buccal swab samples were kept dry and protected from light at room temperature. The first swab was used for the isolation of DNA, while the second swab was kept in the aforementioned conditions for later use if required.

### Extraction of the genomic DNA

DNA was extracted from the buccal mucosa cells using a commercial kit for genomic DNA isolation (Nexttec™ 1-step DNA Isolation; Nextec Applications, Inc., Greenwich, CT, USA). The commercial kit included all the reagents required for the lysis of the cells and purification of the DNA. The aim of the protocol was to retain the proteins, detergents and components of low molecular weight on the resin column, allowing the passage of the DNA through the column. The DNA extracted from the buccal swab samples was preserved in a freezer at −20°C.

### Quality control and semi-quantitation of DNA

The concentration and quality of the DNA obtained using the isolation kit purchased from Nextec Applications, Inc. was analyzed by gel electrophoresis in 1.5% agarose at 80 V for 2 h. Subsequently, the gel was stained with ethidium bromide and the bands were displayed on an ultraviolet transilluminator (FOTO/UV^®^ 21 FOTODYNE 312 nm DNA transilluminators; Fotodyne Incorporated, Hartland, WI, USA). A molecular weight marker was used in each run. The isolated DNA concentration was determined by comparing the fluorescence intensity between the weight marker of a known concentration and DNA bands of unknown concentration. The integrity of the bands was the parameter used to evaluate the quality of the isolated DNA.

### Selection of primers and nucleotide sequence

The single-strand conformational polymorphism (SSCP) technique was employed to analyze exons 2, 3, 5, 20 and 11 of the BRCA1 gene. Considering the large size of exon 11, and in order to analyze it in its entirety, the exon was divided into 12 overlapping fragments. The primers were selected according to a previous study in the Brazilian literature investigating the frequency of mutations in these regions of the BRCA1 gene in females of the Latino population (6).

### Fragment amplification by polymerase chain reaction (PCR)

Each PCR was performed using 50 ng of DNA, 1X PCR Buffer with 1.5 mM MgCl_2_ (Amersham Biosciences, Piscataway, NJ, USA), 200 μM dNTPs (Amersham Biosciences), 10 μM of each primer (see Table I) and 1 unit of Taq DNA polymerase (Amersham Biosciences) in a final volume of 12.5 μl. The PCR protocol used was the same for each amplified gene region with the exception of the annealing temperature, which was specified by each primer pair. The amplification conditions were as follows: Initial denaturalization for 5 min at 96°C; 35 cycles of 30 sec at 96°C and 30 sec at the annealing temperature (annealing) of each pair of primers; 1 min for elongation at 72°C; and a final extension for 10 min at 72°C. The samples were maintained at 4°C until removal from the thermocycler.

### SSCP fragment separation in denaturing polyacrylamide gel

The PCR products were diluted 3:1 in 3X Loading buffer (0.5 M EDTA, 95% formamide and 0.05% bromophenol blue; Promega Corporation, Madison, WI, USA). The PCR products denatured at 98°C for 10 min and immediately placed on ice to allow the fragments of ssDNA to fold into three-dimensional structures as a result of intrastrand base pairing. The gel running conditions were as follows: 700 V and 17 Watt for 11 h at room temperature. To visualize the resulting band pattern of the electrophoretic run, the gel was stained with silver nitrate and revealed with sodium carbonate.

### Polymorphism confirmation

All polymorphisms detected in the acrylamide gel were confirmed by repeating the PCR reaction, subsequently employing the genetic material isolated from the preserved second swab. Next, the reaction was repeated using DNA obtained from the peripheral blood of each patient to sequence and determine the type of mutation existing in the polymorphic region.

### Sequencing of the polymorphism SSCP products

The PCR products with abnormal bands (polymorphisms) in the electrophoretic pattern of the SSCP were sent to Ruralex Fagos (Buenos Aires, Argentina) for sequencing. The sequencing was performed using the dideoxy method using the terminal chain method and Big Dye^®^ technology (Applied Biosystems, Foster City, CA, USA). The primers used for sequencing were the same as those used for the PCR reaction. The cycling conditions were as follows: 96°C for 5 min; 35 cycles of 30 sec at 94°C, 30 sec at 51°C and 4 min at 60°C; followed by a cycle of 10 min at 60°C. The amplification products were purified using a protocol based on MgCl_2_/ethanol and run on an ABI 310 genetic analyzer (Applied Biosystems). The results were analyzed using ABI PRISM^®^ 3100-Avant and 3100 Data Collection v2.0 software (Applied Biosystems).

## Results

**Figure 1:**
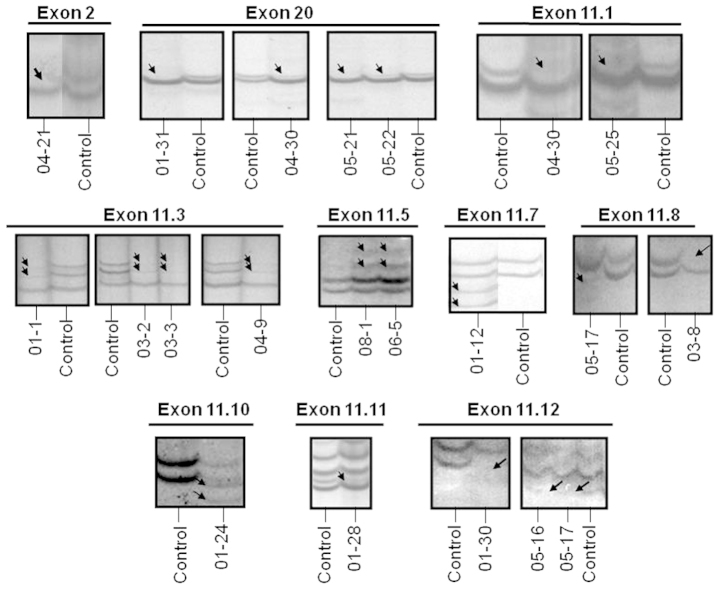
BRCA1 gene polymorphisms detected in 21 out of 154 breast cancer patients using the SSCP method. Images of the SSCP products run in the polyacrylamide non-denaturing gel and developed with silver nitrate. BRCA1 polymorphisms were identified in the following exons of the breast cancer patients (identification number indicated in the figure): 2, 20, 11.1, 11.3, 11.5, 11.7, 11.8, 11.10, 11.11 and 11.12. In each gel, the control column was run in parallel with the sample, corresponding to a normal healthy patient without breast cancer. SSCP, single-strand conformational polymorphism.

**Figure 2:**
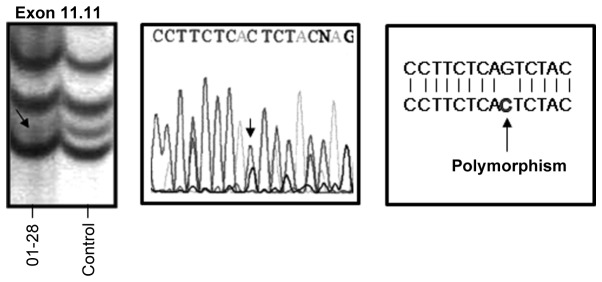
cDNA sequencing of exon 11.11 of the BRCA1 gene. The SSCP-polyacrylamide gel electrophoresis was compared with the SSCP product sequencing of BRCA1 exon 11.11 obtained from patient 28 at the South Regional Italian Hospital (Bahìa Blanca, Argentina). The left figure shows a polymorphism in BRCA1 exon 11.11 which was not identified in the control, the central figure shows the nucleotide sequencing of the SSCP region and the right figure shows the polymorphism identified in the nucleotide 3,674 from exon 11.11 of the BRCA1 gene, corresponding to a single change from guanine to cytosine. SSCP, single-strand conformational polymorphism.

Analysis was performed on a total of 154 DNA samples obtained from females with breast cancer, with or without a family history. Exons 2, 3, 5, 20 and 11 (including the 11.1 to 11.12 regions) of the BRCA1 gene were analyzed. These exons were selected as several studies have shown increased mutagenic frequency in these regions (13–18). Following sampling of the buccal mucosa and subsequent extraction of the DNA as previously described, the SSCP technique was performed based on the separation of the DNA fragments according to their three dimensional conformation in non-denaturing polyacrylamide gels. The DNA of an individual without breast or ovarian cancer was used to identify the altered electrophoretic pattern in patients with cancer. In the full analysis, 21 possible polymorphisms were detected; one in exon 2, four in exon 20, two in exon 11.1, four in exon 11.3, two in exon 11.5, one in exon 11.7, two in exon 11.8, one in exon 11.10, one in exon 11.11 and three in exon 11.12 (Fig. 1, Table II). Overall, 76.20% of the altered electrophoretic activity was identified within exon 11, as predicted given its large size. Of these possible polymorphisms, three were confirmed by performing the full analysis process using the second preserved swab. Subsequently, peripheral blood was extracted from the patients whose results had shown an altered electrophoretic pattern. The DNA was extracted and the samples were sent for sequencing. The samples were sequenced and two indeterminate results were obtained; one a missense mutation of a G to C change at position 3,674 corresponding to exon 11.11 of the BRCA1 gene (Fig. 2).

## Discussion

It has been reported that ~0.1% of the population possess a total of 5,000 different BRCA gene mutations (19). Collaboration among medical professionals and researchers is required to gain increased knowledge concerning the mutational spectrum and ethnic distribution of the different mutations (1). It is also important to establish the pattern of breast cancer risk in the population associated with a given mutation. This may clarify the mechanism responsible and allow the proper precautions to be taken (14).

It is important to identify females at high risk of developing breast cancer. Inheriting a deleterious BRCA mutation is one of the most important predictors of individual risk (15). Only 10% of all breast cancers are hereditary and, as previously described, <1% of the population carry BRCA gene mutations. BRCA mutations are associated with a relative increase of 2.7–6.4 times the risk of breast cancer, as well as an increased risk of ovarian cancer of 9.3–35.3 times the average risk. Females who carry a deleterious mutation in the BRCA genes may be offered chemoprevention, such as early surveillance and prophylactic surgery. Health professionals should increase patient understanding of the risk of mutations in the BRCA genes. It is also important to promote public policies through adherence of the female population to preventive strategies, thus reducing the morbidity and mortality associated with hereditary cancers (20).

As previously described, being a carrier of a BRCA gene mutation is associated with an increased risk of certain types of cancer, particularly breast and ovarian cancer. However, individuals with a family history of BRCA mutations may not carry the mutation and a negative result may be misleading. Therefore, in this study, patients suffering from cancer were employed as the priority group, as these patients have an increased chance of having a BRCA1 gene alteration than healthy females without breast cancer. Although the results of genetic analysis may be normal or without alterations, the patient may have mutations in other regions of the gene which were not analyzed or in other genes associated with the BRCA genes. Notably, the presence of mutations in the BRCA genes in females with primary breast cancer indicates a higher chance of developing a second cancer in another organ, bilateral breast cancer or recurrence of the cancer in the same breast. In addition, healthy relatives may also carry the same genetic alteration in the BRCA1 gene and, therefore, have a significantly higher risk of developing the disease than the general population.

A recent study has yielded encouraging results on the development of a test gene expression profile from the peripheral blood for the early detection of breast cancer with a prediction accuracy of 79.5%, sensitivity of 80.6% and specificity of 78.3% (21).

It is extremely important to perform genetic analysis for BRCA mutations in patients at a high risk of developing breast cancer in Argentina to aid health professionals in addressing the prevention, treatment and prognosis of the disease (1,4). However, such studies have been limited to a small sample size in BRCA1 mutation analysis and, therefore, the current study performed this screening with a large sample size of 154 patients.

The social impact of this study could be important as, to the best of our knowledge, similar studies have not be performed in Argentina. The majority of the studies conducted in different countries differ in their results for the pathological features of patients with breast cancer associated with the type of BRCA1 gene mutation. However, these tumors tend to be of high grade, and less frequently express the estrogen receptor (ER) or progesterone receptor (PR), which have been associated with a poor prognosis in sporadic tumors. The aim of the present study was to determine whether there is a correlation between the type of alteration found in BRCA1 and the histological subtype, which may also be useful for determining the association between the expression of hormone receptors, such as ER, PR and HER2, that were analyzed in all patients included in this study. In addition, this study is important to report the genetic variations in Argentinian females and to examine similarities with the world population.

The social impact of this project may also be important for the prevention and early diagnosis of breast cancer, which are essential to patients carrying mutations in the BRCA 1/2 gene, to select the best and most appropriate method of prevention for the development of breast cancer, including prophylactic breast surgery or risk reduction strategies. It is also important to note that a healthy person possessing a mutation may not develop the disease, but is at a higher risk of developing breast cancer. At present, the use of prophylactic surgery in Argentina is unlikely as the family history of alterations in the BRCA 1 and 2 genes remain unknown. We consider it essential that proper and adequate explanations are provided to BRCA gene mutation carriers with regard to the risk of cancer to facilitate the testing of relatives for genetic alterations and identification of BRCA1 mutation carriers, and subsequently, with the aid of professionals, to select the most appropriate preventive option.

In conclusion, the current study identified 21 polymorphisms in the 154 patients analyzed (14.49%). One patient was identified with a polymorphism in exon 2 (0.69%), four in exon 20 (2.76%), two in exon 11.1 (1.38%), four in exon 11.3 (2.76%), two in exon 11.5 (1.38%), one in exon 11.7 (0.69%), two in exon 11.8 (1.38%), one in exon 11.10 (0.69%), one in exon 11.11 (0.69%) and three in exon 11.12 (2.07%). Polymorphisms are verified by sequencing to determine the type of mutation that characterizes them. The most prevalent polymorphisms of the BRAC1 gene in Argentinian patients were located in exon 11 (16 out of the 21 patients; 76.20%; Table II). The objective of our next study is to evaluate the genetic susceptibility of healthy patients, as well as relatives of BRCA1-positive patients in Argentina, and to analyze 20 regions of the BRCA2 gene.

## Figures and Tables

**Table I:** BRCA1 gene primer nucleotide sequences for single-strand conformational polymorphism analysis.

Exon no.	Nucleotide sequence	Annealing temperature, °C
2	F: GAA GTT GTC ATT TTA TAA ACC TTT	57
	R: TGT CTT TTC TTC CCT AGT ATG T	57
3	F: TCC TGA CAG AGC AGA CAT TTA	53
	R: TTG GAT TTT CGT TCT CAC TTA	53
5	F: CTC TTA AGG GCA GTT GTC AG	58
	R: TTC CTA CTG TGG TTG CTT CC	58
20	F: ATA TGA CGT GTC TGC TCC AC	57
	R: GGG AAT CCA AAT TAC ACA GC	57
11.1	F: CCA AGG TGT ATG AAG TAT GT	57
	R: GAT CAG CAT TCA GAT CTA CC	57
11.2	F: CTC ACT AAA GAC AGA ATG	56
	R: CTT TCT GAA TGC TGC TAT	56
11.3	F: CAG AAA CTG CCA TGC TTC AGA	56
	R: AGG CTT GCC TTC TTC CGA TA	56
11.4	F: GTT CAC TCC AAA TCA GTA GAG AG	56
	R: CAG CTT TGC TTT TGA AGG CAG	56
11.5	F: CCT AAC CCA ATA GAA TCA CTC G	56
	R: GAA CCA GGT GCA TTT GTT AAC TTC	56
11.6	F: CAG CGA TAC TTT CCC AGA GC	56
	R: GTC CCT TGG GGT TTT CAA A	56
11.7	F: CTG GAA GTT AGC ACT CTA GG	56
	R: GTT GCA CAT TCC TCT TCT GC	56
11.8	F: CCG TTT TCA AAT CCA GGA AA	56
	R: TGA TGG GAA AAA GTG GTG GT	56
11.9	F: GAG GCA ACG AAA CTG GAC TCA	56
	R: CTC AGG TTG CAA AAC CCC TA	56
11.10	F: AAC AGA GGG CCA AAA TTG AA	56
	R: GGG TGA AAG GGC TAG GAC TC	56
11.11	F: AAA GCG TCC AGA AAG GAG AGC	56
	R: GCC TTT GCC AAT ATT ACC TGG	56
11.12	F: CAT TGA AGA ATA GCT TAA ATG	56
	R: CCT GGT TCC AAT ACC TAA GTT	56

F, forward; R, reverse.

**Table II:** Prevalence of BRCA1 gene polymorphisms detected in 154 breast cancer patients from the south region of Argentina.

	Polymorphisms detected	
	
Exon no.	Patients with alterations, n	General prevalence, %
2	1	0.69
20	4	2.76
11.1	2	1.38
11.3	4	2.76
11.5	2	1.38
11.7	1	0.69
11.8	2	1.38
11.10	1	0.69
11.11	1	0.69
11.12	3	2.07
Total	21	14.49
